# Morphological Comparisons of Adult Worker Bees Developed in Chinese and Italian Honey Bee Combs

**DOI:** 10.3390/insects16010104

**Published:** 2025-01-20

**Authors:** Shunhua Yang, Hui Li, Pingqing Wu, Dan Yue, Yulong Guo, Wenzheng Zhao, Kun Dong

**Affiliations:** Yunnan Provincial Engineering and Research Center for Sustainable Utilization of Honey Bee Resources, Eastern Bee Research Institute, College of Animal Science and Technology, Yunnan Agricultural University, Kunming 650201, China; fengxue_20141011@163.com (S.Y.); 13769308931@163.com (H.L.); yuedan2015@126.com (D.Y.);

**Keywords:** *Apis cerana*, *Apis mellifera*, comb cell, worker bees, bee morphology

## Abstract

The size of comb cells is a key factor influencing the body size of honey bee workers. Comb cells and the body size of Chinese honey bee workers are smaller than those of Italian honey bee workers. To increase the size of Chinese honey bee workers, this study used newly built combs from Chinese honey bee colonies (control group) and Italian honey bee colonies (treatment group). These combs were provided to Chinese honey bee colonies for queens to lay fertilized eggs with the aim of rearing larger workers. Workers emerging from the control and treatment combs were designated as control and treatment workers, respectively. We compared 13 external morphological traits, including right forewing length and width; linear length of veins a, b, c, and d; proboscis length; right hind femur length; tibia length; metatarsal length and width; and the longitudinal diameters of the third and fourth tergites between the two groups. The results identified six types of cell contents in the combs, excluding empty cells: capped honey (most abundant), followed by capped brood, uncapped honey, and smaller amounts of pollen, larvae, and eggs. Additionally, the average body weights of 6-day-old worker bee larvae, white-eyed pupae, adult worker bees, and honey stomachs containing sucrose solution were significantly higher in the treatment group than in the control group. Except for proboscis length, the average size of 12 out of 13 traits in the treatment group was significantly larger than in the control group, indicating that increasing cell size can produce larger Chinese honey bee workers. In the control group, only the left forewing was significantly shorter than the right, with no significant side-to-side differences in the other morphological traits. In the treatment group, the left forewing width, hind leg femur length, tibia length, and hind leg metatarsus width were significantly smaller than their right-side counterparts, while the other six traits showed no significant side-to-side differences. These findings provide a basis for using Italian honey bee combs to rear larger workers in Chinese honey bee colonies, offering beekeepers a strategy to enhance foraging capacity and improve honey production.

## Simple Summary

Comb cell size is a key factor influencing the body size of honey bee workers. Chinese honey bee workers have smaller comb cells and body sizes than Italian honey bee workers. In this study, newly built combs from Chinese honey bee colonies (control group) and Italian honey bee colonies (treatment group) were provided to Chinese honey bee colonies, allowing the queen to lay fertilized eggs in the cells to rear larger workers. Workers emerging from the control and treatment combs were designated as control and treatment workers, respectively. We compared 13 external morphological traits, including right forewing length and width; linear length of veins a, b, c, and d; proboscis length; right hind femur length; tibia length; metatarsal length and width; and the longitudinal diameters of the third and fourth tergites between the two groups. The results revealed that except for proboscis length, all other measured traits were significantly larger in the treatment group than in the control group, suggesting that increasing cell size directly increases worker body size. This study demonstrates that enlarging comb cell size can produce larger Chinese honey bee workers, providing a theoretical basis for rearing larger Chinese honey bee colonies using Italian worker combs.

## 1. Introduction

The difference in bee body size exists both within and between bee colonies [1,2,3]. Numerous irregular cells exist on natural honey bee combs, functioning as transitional cells between worker and drone cells [4]. Queens lay fertilized eggs in these transitional cells, which then develop into workers [5], leading to variations in worker body sizes within colonies. Bee larval growth increases exponentially [6,7] until each larva fills its developmental cell. Within certain limits, adult workers’ body size matches the cell size where they grow and develop [8]. Thus, bee body size depends on cell size; workers reared in larger cells emerge as large bees, whereas those in smaller cells emerge as small bees [9]. Studies indicate that bee body size metrics, including wing length and width, vein length, proboscis and leg lengths, and other morphological traits, increase proportionally with cell size [3]. A study showed that workers developed in larger cells measuring 5.85 mm in diameter had greater birth weights and body sizes than those in smaller cells measuring 5.65 mm. Larger workers constructed larger cells, which enhanced honey yield by 12–21% [10]. Similarly, workers from 5.85 mm diameter cells in a treatment group show 10.8–20.5% higher birth weights than those from 5.65 mm diameter cells in a control group. Colonies with large bees exhibit stronger overwintering abilities (e.g., reduced rectal fecal accumulation) than control colonies. They also expand queen cell openings to 11–12 mm, resulting in an 8.4% increase in queen birth weight [11]. Bees reared in 5.85 mm diameter cells attained 10% higher honey yields and 25% more combs than those in 5.65 mm cells [12]. In summary, large worker bees reared from large cells outperform small bees in production efficiency.

The differences in bee body size may affect the labor division. The labor division in bees is related to age; young house bees handle brood rearing, honey production, and other in-nest tasks, later transitioning to foraging, propolis collection, and other field tasks as they age [13]. However, worker bee body size affects the age at which they begin foraging. For instance, heavier, large-bodied bumblebee workers, like those of *Bombus agrorum*, become foragers earlier than lighter, medium-bodied workers, while the lightest, small-bodied workers may remain in the hive their entire lives [14]. The division of labor among *Apis* bees, specifically *Apis mellifera*, is also influenced by body size. Larger worker bees start more advanced tasks in development at younger ages than smaller ones. Even small differences in body size affect the age at which worker bees begin foraging, transitioning from hive to foraging activities [15]. Compared to smaller foragers, larger ones have bigger honey stomachs, allowing an average nectar load ratio to an unloaded weight of 82% [16]. They also carry more pollen due to larger pollen baskets [17,18,19] and make more frequent and longer trips, although trip duration does not vary with body size [1,20,21]. Additionally, variations in honey bee worker body size influence dance communication efficacy. As social insects, honey bees rely on food recruitment systems, which necessitate small body size differences to maintain accuracy in recruitment signals [22]. Therefore, worker bee body size must remain similar to ensure precise information transmission.

As part of the bee recruitment dance, two factors depend at least partially on bee body size: the distance from the hive to the flower and flower profitability. Distance is measured based on energy expenditure [23,24], which varies with body size. Profitability relies on the nectar amount and concentration obtained from flowers and handling and flight costs between flowers [25,26], which may also vary with body size. Thus, information transfer between scout and recruit bees of different sizes may not reflect what recruits will experience upon reaching flowers. For example, larger scout bees may gain higher profits from flowers with long corollas and abundant nectar. The scout signals “high profitability” to dance followers regardless of size. However, this information only applies to similarly sized recruits. Smaller recruits may incur higher handling costs and may not harvest all nectar from a flower. Spreading inaccurate information can be costly if bees are recruited to flowers inefficiently for foraging. Misinformation increases with greater body size variation, reducing recruitment system efficiency [27]. Thus, before complex recruitment systems could evolve, colonies needed less body size variation. Bees with recruitment systems may have also developed behavioral mechanisms to mitigate the negative effects of body size variation [22]. Research on 31 bee species shows that honey bees (*Apis*) exhibit the smallest body size variation [28].

For bees, many economically valuable traits related to product yields, such as honey, propolis, and pollen production, can only be assessed at the colony level and are affected by both internal and external environmental factors. Therefore, directly determining measurement accuracy for these traits is challenging. For instance, honey production can only be assessed during the flowering period of nectar-producing plants, making it highly influenced by environmental conditions. The limited evaluation period for various traits has delayed advancements in honey bee improvement programs. Thus, to enhance bee product yield, researchers have identified production-relevant traits that can be easily measured, enabling indirect selection methods for breeding high-yield colonies and bypassing lengthy selection processes. Currently, there are two methods for assessing bee production. The first involves standardizing environmental conditions across all colonies to measure production at the colony level. The second assesses colonies based on traits, such as morphological characteristics, that are minimally influenced by the environment and closely associated with production [29]. The genetic estimate for these traits is high [30,31], with Africanized honey bees being smaller in size than European bees [30]. Under similar conditions, Africanized honey bees tend to store less honey than European honey bees [32].

Morphological traits used to evaluate bee production performance include proboscis length [33], pollen basket area [17], wing length [34], and hind leg length [35]. The worker bee proboscis length indicates its nectar-sucking potential; a longer proboscis enhances access to nectar within deep corollas. The third and fourth tergite lengths reflect worker bee body size, which correlates with honey stomach volume. A longer tergite suggests a larger body size and greater nectar storage capacity. Forewing size in worker bees reflects flight ability, where larger wings enhance flight strength, speed, and collection area [36]. The Chinese honey bee is a subspecies of the Eastern honey bee, while the Italian honey bee is a subspecies of the Western honey bee; reproductive isolation prevents hybridization between these species [37]. The average cell diameter of Chinese worker bees is 11.6% smaller than that of Italian worker bees, while Chinese worker bees are 1.86 mm shorter in average body length than Italian worker bees, making their body length about 86% of that of Italian worker bees. Additionally, the proboscis of Chinese worker bees averages 1.20 mm shorter than that of Italian worker bees, making it about 81% as long [38]. The stomach capacity of Chinese worker bees is smaller than that of Italian worker bees, and the average nectar load of each Chinese worker bee is 5.20 mg less than that of Italian honey bee workers. The average weight of pollen pellets carried by Chinese worker bees is 14.5 mg, accounting for 19.3% of their body weight, whereas the average pollen pellet weight carried by Italian worker bees is 17.5 mg, representing 18.1% of their body weight [39]. In other words, the pollen load of Chinese bees is only 83% of the Italian bees’ load. Italian worker bees can collect nectar from deep corolla flowers, whereas Chinese bees, with their shorter proboscises, cannot access deep corolla nectar. The diameter of Chinese worker bee cells is smaller than that of Italian bee cells, resulting in the development of smaller worker bees with reduced honey stomach volume, nectar collection volume, and a slower rate of nectar intake by the colony. Conversely, the larger cell diameter in Italian worker bees allows for the development of larger workers with greater honey stomach volume, enhanced nectar collection volume, and faster nectar intake. To increase the nectar intake of Chinese honey bee colonies, a method is needed to boost individual nectar collection. Although directly enlarging the honey stomach volume of worker bees is impossible, it is feasible to indirectly enhance nectar collection by rearing larger individuals.

In this study, large-sized Chinese worker bees were successfully reared by enlarging cell size. This provides a theoretical basis for Chinese honey bee colonies to rear large worker bees using Italian worker combs. It also offers guidance for beekeepers to develop efficient breeding strategies, improve the collection potential of Chinese honey bees, and achieve high-quality, high-yield bee products. Therefore, the following hypotheses were proposed in this study: (1) with abundant nectar sources, the cell serves as a development site; the Italian worker bee cell is larger than the Chinese worker bee cell, allowing Chinese honey bee queens to lay fertilized eggs in Italian worker bee cells where they develop into worker bees; (2) body size differences exist between adult worker bees reared in Italian and Chinese worker bee cells; (3) worker cells of Italian or Chinese honey bees affect the body symmetry of Chinese worker bees.

## 2. Materials and Methods

### 2.1. Establishing Experimental Bee Colonies to Rear Worker Bees

**Figure 1:**
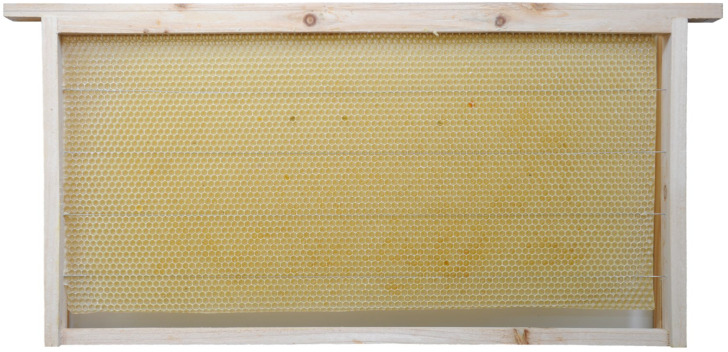
Newly built Chinese worker bee comb with a wax foundation.

**Figure 2:**
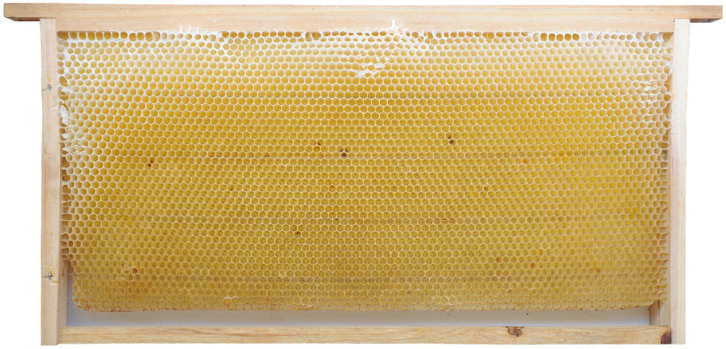
Newly built Italian worker bee comb with a wax foundation.

Seven Chinese honey bee (*Apis cerana cerana*) colonies with similar colony strength were used as experimental colonies. Each queen was a first-time, successfully mated, egg-laying queen. Each hive contained four combs initially. After removing two combs from each hive, a newly built Chinese worker bee comb (Figure 1) with a wax foundation created by a Chinese honey bee colony was added as a control comb. Additionally, a newly built Italian worker bee comb (Figure 2) with a wax foundation made by an Italian honey bee (*Apis mellifera ligustica*) colony was added as a treatment comb for the Chinese honey bee queen to lay eggs, rear broods, and store food. The cell base diameters of Chinese and Italian worker bee wax foundations were 4.75 ± 0.0030 mm (N_cell base_ = 60, N_comb foundation_ = 6) and 5.34 ± 0.0031 mm (N_cell base_ = 60, N_comb foundation_ = 6), respectively. The development of broods on the experimental combs was checked every 3 days, and appropriate numbers of 6-day-old worker larvae (N_larvae_ = 600, N_colony_ = 6) and white-eyed worker pupae (N_larvae_ = 600, N_colony_ = 6) were collected and weighed. When capped broods on experimental combs were close to emerging, the combs were removed, and nurse bees on the comb surface were quickly stripped off. A camera was used to photograph both sides of the experimental combs. The photographed experimental combs were placed into queen oviposition control, and the grid size was adjusted to prevent workers from escaping. The queen oviposition controllers, containing the experimental combs, were then placed in a constant temperature and humidity incubator (temperature 35 ± 0.1 °C, relative humidity 75 ± 0.1%) for incubation until broods developed into adults and emerged from cells [40].

### 2.2. Counting Cell Contents

According to the photos of experimental combs described in Section 2.1, the percentage of cells containing different contents and the total cell count in each comb were calculated. The main cell contents included capped honey, uncapped honey, pollen, sealed broods, larvae, and eggs. Some cells on the combs remained empty.

### 2.3. Measurement of Birth Weight and Morphological Indicators of Emerging Worker Bees

**Figure 3:**
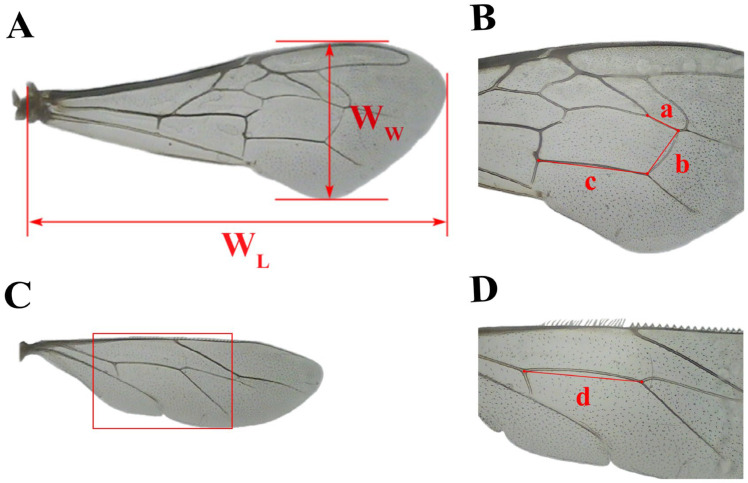
Right forewing and right hindwing of a Chinese worker bee. (**A**) Forewing length W_L_ and width W_W_; (**B**) linear lengths of wing veins a, b, and c; (**C**) hind wing; and (**D**) hind wing vein d.

**Figure 4:**
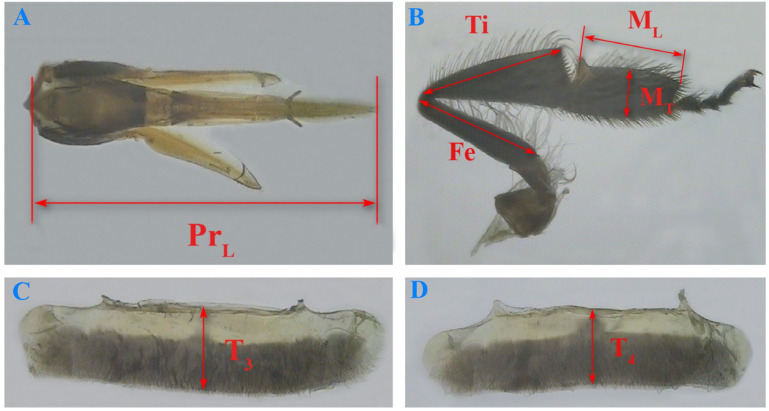
The four key morphological characteristics of the Chinese worker bee. (**A**) Proboscis length Pr_L_; (**B**) the length of the right hind leg femur Fe, tibia Ti, metatarsus M_L_, and the width of the right hind leg metatarsus M_T_; (**C**) longitudinal diameter of the third tergite T_3_; and (**D**) the fourth tergite T_4_.

As soon as the sealed broods emerged from the cells, the birth weight of the worker bees was recorded immediately with an electronic balance. Weighed worker bees were then divided into groups A (N_bee_ = 786, N_colony_ = 7) and B. Group A (N_bee_ = 360, N_colony_ = 6) was subjected to measurement for honey stomach capacity, and group B was used to measure external morphological dimensions. Honey stomach capacity was measured by feeding worker bees a sucrose solution with 50% sucrose content, then quickly dissecting the sucrose-filled honey stomachs from the abdominal cavity and weighing them. Forewing length, forewing width, linear lengths of forewing veins a, b, and c, and linear length of hindwing vein d (Figure 3); proboscis length; lengths of hind leg femur, tibia, and metatarsus; width of hind leg metatarsus; and longitudinal diameters of the third and fourth tergites (Figure 4) served as indicators for measuring the external morphological characteristics of worker bees that emerged from different brood cell sizes [41].

Live worker bees were placed in water at 60 °C, and after their proboscis was fully extended, the proboscis, wings, hind legs, and third and fourth tergites were carefully removed with tweezers and placed on glass slides. A charge-coupled device (CCD) video microscope, produced by Shenzhen Zhongzheng Instrument Co., Ltd. in Shenzhen, China, was used to capture images of the scale and the morphological indicators. Bee morphometry software version 1.01 was used to measure the size of each morphological feature, and the scale’s precision was ±0.01 mm.

### 2.4. Statistical Analysis

Statistical analyses were conducted using GraphPad Prism 9.5 software (GraphPad Software, San Diego, CA, USA). The Kolmogorov–Smirnov test was used to determine the normal distribution of experimental data in each group. An unpaired *t*-test was applied to indicators with normal distribution and homogeneous variance, including body weight of 6-day-old larvae, bodyweight of white-eyed pupae, right forewing width, linear lengths of forewing veins a, b, and c, linear length of hindwing vein d, right hind leg metatarsus width, and fourth tergite longitudinal diameter. Welch’s *t*-test was performed on indicators with normal distribution but heteroscedasticities, such as honey stomach capacity, birth weight, proboscis length, right forewing length, right hind leg femur, right hind leg tibia, right hind leg metatarsus, third tergite longitudinal diameter, and cell diameter. Paired *t*-tests were used on indicators with left-right symmetry (wings, wing veins, legs), with a significance level of α = 0.05, and statistical values are reported as mean ± standard error. Pearson correlation analysis was conducted to examine the associations between worker body weight, specific morphological traits, and cell diameter.

## 3. Results

### 3.1. Comparison Between Cell Diameters of Chinese Worker Bees and Italian Worker Bees

The average cell diameter of Chinese worker bees (4.72 ± 0.008 mm, N_cell_ = 30) was significantly smaller than that of Italian worker bees (5.32 ± 0.014 mm, N_cell_ = 30), as determined by Welch’s *t*-test (t = 37.28, df = 44.68, *p* < 0.0001).

### 3.2. Percentages of Cells with Different Contents

**Figure 5:**
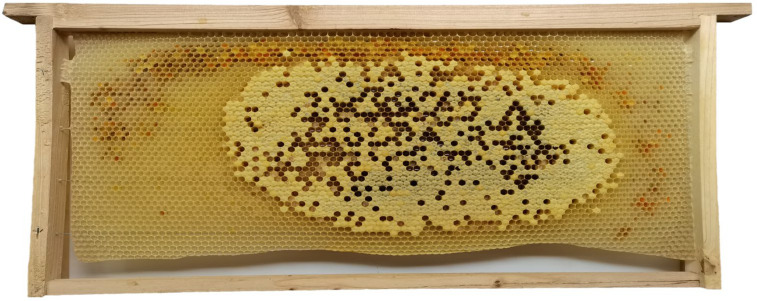
A sealed brood comb from the control group.

**Figure 6:**
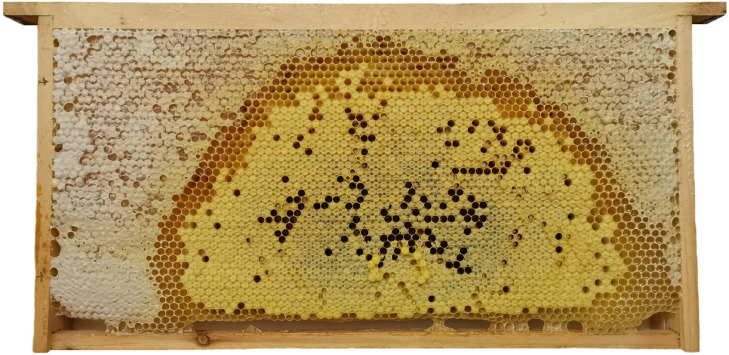
A sealed brood comb from the treatment group.

**Figure 7:**
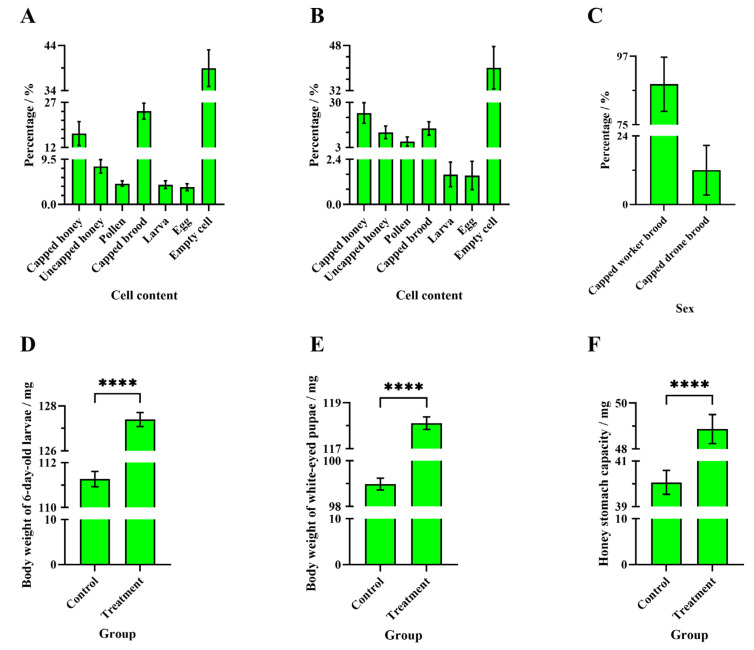
The percentages of cells with different contents in the brood comb and the three weight indicators: (**A**) control group, (**B**) treatment group, (**C**) the percentages of worker and drone-capped broods on the brood comb of the treatment group, and body weights of (**D**) 6-day-old larvae, (**E**) white-eyed pupae, and (**F**) honey stomach capacity. The asterisk indicates significant differences (****, *p* < 0.0001).

In addition to empty cells, cells in the experimental combs in both the control group (Figure 5) and the treatment group (Figure 6) contained capped honey, uncapped honey, pollen, sealed brood, larvae, and eggs. In the control group combs, the average percentages of cells containing capped honey, uncapped honey, pollen, sealed brood, larvae, and eggs relative to the total cell count were 16.65 ± 3.98%, 8.04 ± 1.43%, 4.41 ± 0.57%, 24.12 ± 2.66%, 4.19 ± 0.82%, and 3.64 ± 0.73%, respectively (Figure 7A). In the treatment group combs, the average percentages for capped honey, uncapped honey, pollen, sealed brood, larvae, and eggs were 23.66 ± 6.18%, 12.04 ± 3.77%, 6.61 ± 2.76%, 14.43 ± 4.03%, 1.60 ± 0.65%, and 1.54 ± 0.76%, respectively (Figure 7B). In the treatment group combs of the four experimental colonies, not only worker-sealed brood but also drone-sealed brood were present. The average percentage of drone-sealed brood relative to the total sealed brood on the experimental combs was 11.99 ± 8.70% (Figure 7C).

### 3.3. Comparisons of Body Weights of 6-Day-Old Larvae, White-Eyed Pupae, and Honey Stomach Capacity in Worker Bees Between the Two Groups

The average body weight of 6-day-old worker bee larvae in the treatment group (127.40 ± 0.31 mg, N_bee_ = 300) was significantly higher than that in the control group (111.30 ± 0.34 mg, N_bee_ = 300) (unpaired *t*-test, t = 34.94, df = 598, *p* < 0.0001) (Figure 7D). The average body weight of white-eyed worker bee pupae in the treatment group (118.10 ± 0.27 mg, N_bee_ = 300) was also significantly higher than that in the control group (98.98 ± 0.26 mg, N_bee_ = 300) (unpaired *t*-test, t = 51.18, df = 598, *p* < 0.0001) (Figure 7E). Additionally, the average honey stomach weight, filled with sucrose solution, in newly emerged worker bees in the treatment group (48.87 ± 0.63 mg, N_bee_ = 180) was significantly higher than in the control group (40.06 ± 0.52 mg, N_bee_ = 180) (Welch’s *t*-test, t = 10.70, df = 345.6, *p* < 0.0001) (Figure 7F).

### 3.4. Comparisons of Birth Weight, Proboscis Length, and Longitudinal Diameter of the Third and Fourth Tergite Between the Two Groups of Worker Bees

**Figure 8:**
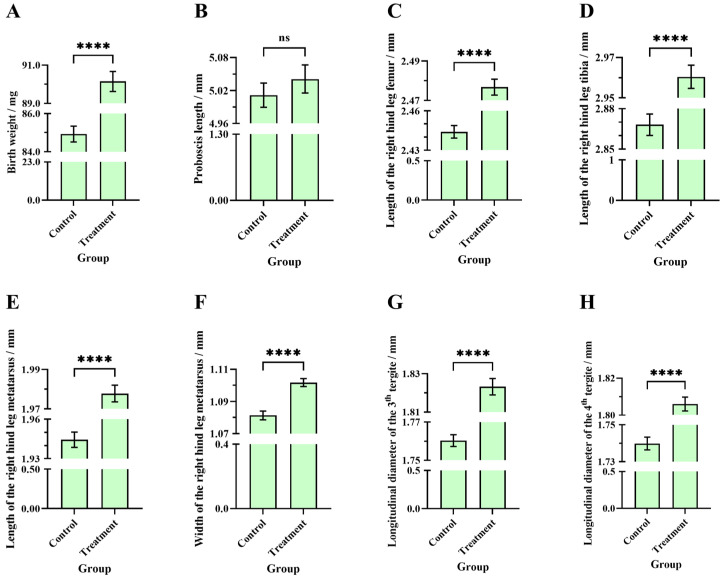
Comparisons of birth weight and morphological indicators between the worker bees from the treatment and control groups: (**A**) birth weight, (**B**) length of proboscis, (**C**) length of the right hind leg femur, (**D**) tibia, (**E**) metatarsus, and (**F**) the width (**G**) longitudinal diameter size of the third and (**H**) fourth tergite. The asterisk indicates significant differences (****, *p* < 0.0001), while “ns” indicates not-significant differences (*p* > 0.05).

The average birth weight of worker bees in the treatment group (90.15 ± 0.53 mg, N_bee_ = 394) was significantly higher than in the control group (84.92 ± 0.41 mg, N_bee_ = 392) (Welch’s *t*-test, t = 7.81, df = 740.3, *p* < 0.0001) (Figure 8A). There was no significant difference in the average proboscis length of worker bees between the treatment (5.04 ± 0.03 mm, N_bee_ = 210) and control groups (5.01 ± 0.02 mm, N_bee_ = 210) (Welch’s *t*-test, t = 0.8726, df = 408.1, *p* = 0.38) (Figure 8B). The average longitudinal diameter of the third tergite of worker bees in the treatment group (1.82 ± 0.0042 mm, N_bee_ = 210) was significantly higher than in the control group (1.76 ± 0.0031 mm, N_bee_ = 210) (Welch’s *t*-test, t = 12.01, df = 379.9, *p* < 0.0001) (Figure 8G). The average longitudinal diameter of the fourth tergite of worker bees in the treatment group (1.81 ± 0.0037 mm, N_bee_ = 210) was significantly higher than in the control group (1.74 ± 0.0034 mm, N_bee_ = 210) (Welch’s *t*-test, t = 13.05, df = 418, *p* < 0.0001) (Figure 8H).

### 3.5. Comparisons of the Length of the Right Hind Leg Femur, Tibia, Metatarsus, and the Metatarsus Width Between the Two Groups of Worker Bees

The average length of the right hind leg femur of worker bees in the treatment group (2.48 ± 0.0041 mm, N_bee_ = 210) was significantly higher than in the control group (2.44 ± 0.0047 mm, N_bee_ = 210) (Welch’s *t*-test, t = 5.22, df = 409.9, *p* < 0.0001) (Figure 8C). The average length of the right hind leg tibia of worker bees in the treatment group (2.96 ± 0.0057 mm, N_bee_ = 210) was significantly higher than in the control group (2.87 ± 0.0080 mm, N_bee_ = 210) (Welch’s *t*-test, t = 9.39, df = 379.9, *p* < 0.0001) (Figure 8D). The average length of the right hind leg metatarsus of worker bees in the treatment group (1.98 ± 0.0042 mm, N_bee_ = 210) was significantly higher than in the control group (1.94 ± 0.0058 mm, N_bee_ = 210) (Welch’s *t*-test, t = 4.66, df = 381.9, *p* < 0.0001) (Figure 8E). The average width of the right hind leg metatarsus of worker bees in the treatment group (1.10 ± 0.0025 mm, N_bee_ = 210) was significantly higher than in the control group (1.08 ± 0.0028 mm, N_bee_ = 210) (unpaired *t*-test, t = 5.49, df = 418, *p* < 0.0001) (Figure 8F).

### 3.6. Comparisons of the Right Forewing Length, Right Forewing Width, and Linear Length of Veins a, b, c, and d Between the Two Groups of Worker Bees

**Figure 9:**
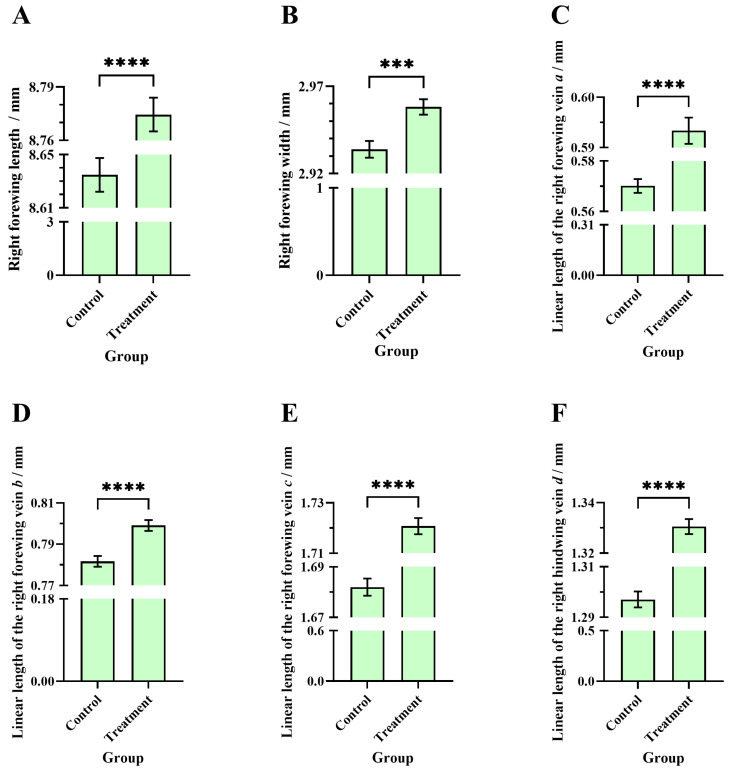
Comparisons of morphological indicators between the worker bees from the treatment and control groups: (**A**) length of the right forewing, (**B**) width of the right forewing, (**C**) linear length of the right forewing vein a, (**D**) b, (**E**) c, and (**F**) linear length of the right hindwing vein d. The asterisk indicates significant differences (***, *p* < 0.001; ****, *p* < 0.0001).

The average right forewing length of worker bees in the treatment group (8.78 ± 0.0095 mm, N_bee_ = 210) was significantly higher than in the control group (8.64 ± 0.01 mm, N_bee_ = 210) (Welch’s *t*-test, t = 8.86, df = 387.9, *p* < 0.0001) (Figure 9A). The average right forewing width of worker bees in the treatment group (2.96 ± 0.0044 mm, N_bee_ = 210) was significantly higher than in the control group (2.93 ± 0.0048 mm, N_bee_ = 210) (unpaired *t*-test, t = 3.75, df = 418, *p* = 0.0002) (Figure 9B). The average linear length of the right forewing vein a of worker bees in the treatment group (0.59 ± 0.0026 mm, N_bee_ = 210) was significantly higher than in the control group (0.57 ± 0.0027 mm, N_bee_ = 210) (unpaired *t*-test, t = 6.15, df = 418, *p* < 0.0001) (Figure 9C). The average linear length of the right forewing vein b of worker bees in the treatment group (0.80 ± 0.0027 mm, N_bee_ = 210) was significantly higher than in the control group (0.78 ± 0.0025 mm, N_bee_ = 210) (unpaired *t*-test, t = 4.74, df = 418, *p* < 0.0001) (Figure 9D). The average linear length of the right forewing vein c of worker bees in the treatment group (1.72 ± 0.0032 mm, N_bee_ = 210) was significantly higher than in the control group (1.68 ± 0.0034 mm, N_bee_ = 210) (unpaired *t*-test, t = 8.29, df = 418, *p* < 0.0001) (Figure 9E). The average linear length of the right hindwing vein d of worker bees in the treatment group (1.33 ± 0.0030 mm, N_bee_ = 210) was significantly higher than in the control group (1.30 ± 0.0031 mm, N_bee_ = 210) (unpaired *t*-test, t = 7.74, df = 418, *p* < 0.0001) (Figure 9F).

### 3.7. Body Side Symmetry in Worker Bees

The results of the paired *t*-test showed that the average left forewing length of worker bees was significantly shorter than the right forewing length in the control group. No significant differences were observed in other indicators between the left and right sides. Although a significant difference was found in left and right forewing length in the control group, Cohen’s d = 0.17 < 0.2, indicating an extremely small effect size of the body side on forewing symmetry (Table 1). The paired *t*-test results showed that in the treatment group, the average width of the left forewing, and the average lengths of the left hind leg femur and tibia, as well as the left hind leg metatarsus width, were significantly smaller than those on the right side. No other significant differences were observed between the left and right sides. Despite significant differences in these four indicators for the treatment group, Cohen’s d < 0.5 indicated a very small effect size of the body side on the symmetry of the forewings, hind leg femur, tibia, and metatarsus (Table 1).

### 3.8. Correlation Analysis Between Worker Body Weight, Morphological Traits, and Cell Diameter

**Figure 10:**
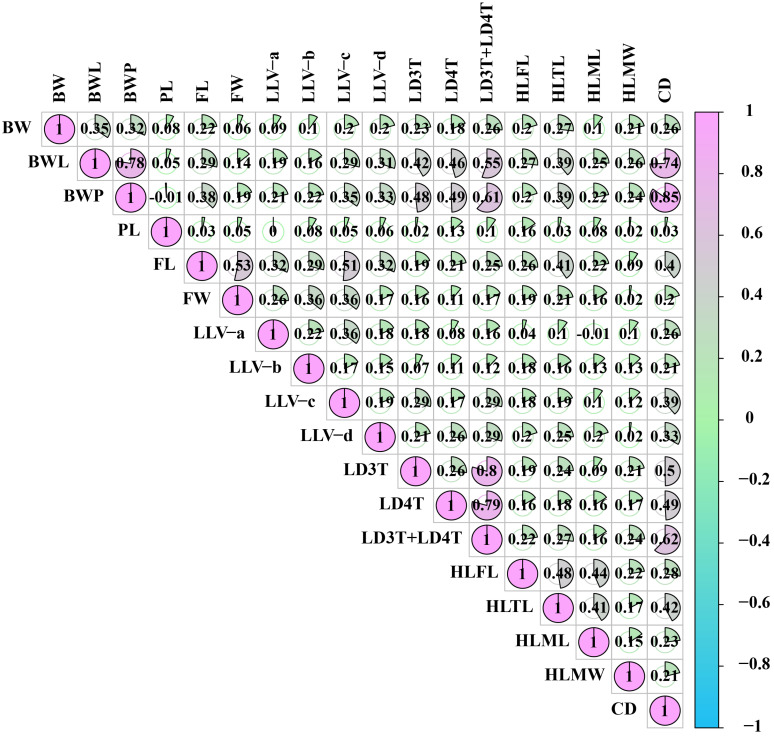
Correlation analysis between worker body weight, morphological traits, and cell diameter. The light green and pink color indicates a positive correlation; the pie chart size indicates the correlation’s magnitude, and the numbers show the correlation coefficient. Birth weight, BW; body weight of 6-day-old larvae, BWL; body weight of white-eyed pupae, BWP; proboscis length, PL; forewing length, FL; forewing width, FW; linear length of vein a, LLV-a; linear length of vein b, LLV-b; linear length of vein c, LLV-c; linear length of vein d, LLV-d; longitudinal diameter of the 3rd tergite, LD3T; longitudinal diameter of the 4th tergite, LD4T; longitudinal diameter of the 3rd + 4th tergite, LD3T + LD4T; hind leg femur length, HLFL; hind leg tibia length, HLTL; hind leg metatarsus length, HLML; hind leg metatarsus width, HLMW; cell diameter, CD.

No significant correlations were found between adult worker bee birth weight and proboscis length, forewing width, or the linear lengths of forewing veins a and b (*p* > 0.05). Likewise, there were no significant correlations between the body weights of 6-day-old larvae, white-eyed pupae, and proboscis length (*p* > 0.05). Similarly, no significant correlations were observed between proboscis length and the linear lengths of veins a, b, c, and d; the longitudinal diameter of the 3rd tergite; hind leg tibia length; the length and width of hind leg metatarsus; or the cell diameter (*p* > 0.05). Additionally, the hind leg metatarsus width showed no correlation with the length and width of forewing (*p* > 0.05), and the linear length of forewing vein a was not correlated with the longitudinal diameter of the 4th tergite, hind leg femur length, or hind leg metatarsus length (*p* > 0.05). There were also no correlations between the linear length of forewing vein b and the longitudinal diameter of the 3rd tergite (*p* > 0.05) or between the linear length of hindwing vein d and the hind leg metatarsus width (*p* > 005). The longitudinal diameter of the 3rd tergite did not correlate with the length of hind leg metatarsus (*p* > 0.05). In contrast, all other relationships showed positive correlations (*p* < 0.05), as shown in Figure 10.

## 4. Discussion

### 4.1. Methods of Rearing Large-Bodied Chinese Worker Bees

Currently, two effective methods exist to increase the body size of Chinese worker bees. The first method involves adding movable frames with wax foundations from Italian honey bees into a Chinese honey bee colony nest. This allows the colony to build combs with cells matching the diameter of Italian worker bee cells. Beekeepers observed that two days after adding the Italian wax foundations, the cells built by the Chinese bee colony were the same size as the Italian worker cells. However, the cell walls gradually thickened, reducing the cell mouth size. This allows the queen bee to lay fertilized eggs, which develop into worker bees in these cells [42]. The second method is the direct use of Italian worker bee combs to rear Chinese worker bees. Beekeeping observations indicate this method works best with colonies that have a newly mated queen actively laying eggs. This is preferable to avoid combining Chinese and Italian worker combs within the hive. In other words, it is more effective to use only Italian worker bee combs to establish a Chinese honey bee colony [43,44]. The queen lays fertilized eggs, which become worker bees, and unfertilized eggs, which develop into drone bees, in the cells of Italian worker bee combs [45,46,47]. Worker bees developing in Italian-sized cells grow larger than those from smaller Chinese bee cells [48]. The average weight of drone pupae of Chinese honey bees reared in Italian worker bee cells reached 148 mg, significantly heavier than those reared on natural Chinese combs [49]. Colonies formed with Italian combs also showed increased resistance to wax moths [50]. In this study, we adopted the second method to experiment with increasing Chinese worker bee body size. The experimental process was straightforward, yielding positive results while providing valuable experience and technical insights for rearing larger-bodied Chinese worker bees. This beekeeping technique has practical potential for Eastern honey bee apiculture.

### 4.2. The Advantages and Significance of Enlarged Chinese Worker Bees

Our results showed that enlarging cell diameters improved the body size of Chinese worker bees. Pollen baskets are specialized hind-leg structures that worker bees use to transport pollen, a primary protein source essential for colony development. Enhanced pollen transport by each worker may result in more field bees collecting nectar, potentially increasing honey yield. Worker bees with larger pollen baskets can carry larger pollen loads, and a greater pollen influx promotes brood rearing, increasing colony size and potential strength. A positive correlation exists between worker bee numbers and honey yield [51]. Thus, colonies with larger-bodied workers may outperform those with smaller-bodied ones. Research on western honey bees indicates that increasing worker body size enhances honey yield [17]. Additionally, the ability of Chinese honey bees to use Italian honey bee combs demonstrates their survival advantage. Conversely, Italian honey bees cannot utilize Chinese honey bee combs.

Large-bodied bees may have higher economic value and ecological significance than small-bodied bees due to productivity, foraging efficiency, and flower handling [52]. For example, worker bees reared in cells with a larger diameter of 5.85 mm showed increases in body weight by 8.66%, honey stomach content by 13.2%, and thorax weight by 4.8% compared to bees reared in smaller 5.45 mm cells. Queens from colonies with large-bodied workers weighed 4.4% more than queens from colonies with small-bodied workers. Honey yield and honey stomach weight in bee colonies using combs with 6.00 mm cells increased by 15.2% and 14.4%, respectively, compared to colonies with 5.45 mm cells [53]. Colonies in combs with large cells (5.65 mm) had honey yields 11.1% to 15.5% higher (averaging 16.9% annually) compared to colonies in 5.40 mm cells [54]. The proboscis length, thorax width, wing length, and honey stomach dimensions in *Apis cerana* workers reared in 5.24 mm cells were 3.70–3.78% larger than those from 4.52 mm cells. Similarly, in *Apis mellifera*, workers reared in 6.00 mm cells showed 9.20–9.29% increases in these traits compared to those from 5.24 mm cells. In both species, workers reared in large cells had heavier bodies, collected more pollen, and lived longer than those from small cells (notably the Eastern honey bee) [55]. Nectar source richness varied seasonally—highest in spring, moderate in summer, and sparse in autumn. This seasonal change increases foraging distances from spring to summer [56], with larger bees flying farther [57,58,59]. Larger bees also have longer legs and larger pollen baskets. In areas with limited nectar sources and long flight distances, colonies rear larger workers to compensate for foraging challenges. These larger workers have bigger wing areas, well-developed flight muscles, and greater foraging ranges [60] and are less prone to predation [56,61].

### 4.3. How Larger Cells Aid in Rearing Larger Bees

The diameter of Chinese worker bee cells based on commercial wax comb foundations on the market is 0.54–0.65 mm smaller than that of Italian worker bee cells. Bee species primarily determine body size, with Italian worker bees having larger bodies than Chinese worker bees. Cell size mainly depends on bee body size and genetics [62]. Thus, increasing cell size only enlarges bees up to a certain range, and once the maximum size is reached, further cell size increases have no effect. Research suggests that large cells can indirectly increase worker body size by altering larval food amounts [11,63,64,65,66], and by providing more or less growth space [63], affecting adult weight and body size. Cell size may also affect food quality, as Gontarski [67] found that cell shape and size determine larval food type, not larval caste. However, nurse bees in queen-right colonies recognize larval sex [68] and feed older larvae of both sexes accordingly [69]. From this perspective, each factor impacting immature and mature bee weight directly or indirectly alters larval food quantity, quality, or both. Changes in bee weight and growth rate, particularly in larvae, may result in differences in actual, physiological, and morphological bee ages [70,71].

Bumblebee genomic studies have revealed potential physiological and behavioral factors linked to body size polymorphisms in workers. For instance, evidence suggests differences in gene expression [72] and RNA editing patterns [73] between small and large worker bees. Similarly, we suggest that body size differences in Chinese worker bees, indirectly caused by cell space, may lead to variations in gene expression and metabolism. Therefore, we plan to study gene expression and non-targeted metabolism in large and small-bodied worker bees in the future.

## 5. Conclusions

Chinese honey bee queens lay many fertilized and few unfertilized eggs in newly built Italian worker cells, producing workers and drones, respectively. The adult workers emerging from Italian worker cells are larger than those from Chinese worker cells. Enlarging cell size can rear larger workers but may increase asymmetry between left and right body sides, although this asymmetry remains minor.

## Figures and Tables

**Table 1:** Paired *t*-test on left and right sides of worker bees between the control and treatment groups.

Group	Index	Mean of Differences (Left–Right)	SD of Differences	95% Confidence Interval	t	df	*p*-Value	Cohen’s d
Control	Forewing length	−0.0095	0.0559	−0.0171 to −0.0019	2.4620	209	0.0146	0.1699
	Forewing width	−0.0031	0.0566	−0.0108 to 0.0046	0.7956	209	0.4272	0.0549
	Linear length of vein a	−0.0009	0.0314	−0.0052 to 0.0034	0.4271	209	0.6698	0.0295
	Linear length of vein b	0.0032	0.0324	−0.0012 to 0.0076	1.4130	209	0.1593	0.0975
	Linear length of vein c	−0.0022	0.0355	−0.0070 to 0.0026	0.9099	209	0.3639	0.0628
	Linear length of vein d	−0.0005	0.0135	−0.0024 to 0.0013	0.5778	209	0.5640	0.0399
	Hind leg femur length	−0.0020	0.0322	−0.0063 to 0.0024	0.8880	209	0.3756	0.0613
	Hind leg tibia length	−0.0076	0.0565	−0.0152 to 0.0001	1.9370	209	0.0541	0.1337
	Hind leg metatarsus length	0.0067	0.0561	−0.0010 to 0.0143	1.7230	209	0.0863	0.1189
	Hind leg metatarsus width	−0.0019	0.0265	−0.0055 to 0.0017	1.0400	209	0.2997	0.0717
Treated	Forewing length	0.0008	0.0306	−0.0034 to 0.0049	0.3641	209	0.7161	0.0251
	Forewing width	−0.0071	0.0428	−0.0129 to −0.0013	2.4030	209	0.0171	0.1658
	Linear length of vein a	0.0017	0.0224	−0.0013 to 0.0048	1.1300	209	0.2596	0.0780
	Linear length of vein b	−0.0004	0.0332	−0.0050 to 0.0041	0.1908	209	0.8489	0.0132
	Linear length of vein c	−0.0006	0.0197	−0.0033 to 0.0021	0.4450	209	0.6568	0.0307
	Linear length of vein d	−0.0012	0.0090	−0.0024 to 0.0000	1.9210	209	0.0560	0.1326
	Hind leg femur length	−0.0049	0.0206	−0.0078 to −0.0021	3.4740	209	0.0006	0.2397
	Hind leg tibia length	−0.0083	0.0379	−0.0135 to −0.0032	3.1940	209	0.0016	0.2204
	Hind leg metatarsus length	−0.0019	0.0374	−0.0070 to 0.0032	0.7429	209	0.4584	0.0513
	Hind leg metatarsus width	−0.0068	0.0258	−0.0104 to −0.0033	3.8370	209	0.0002	0.2648

## Data Availability

The preprocessed data that support the findings of this study are available from the corresponding author upon reasonable request.
